# Immunoglobulin Replacement Therapy in Secondary Hypogammaglobulinemia

**DOI:** 10.3389/fimmu.2014.00626

**Published:** 2014-12-08

**Authors:** Nicolò Compagno, Giacomo Malipiero, Francesco Cinetto, Carlo Agostini

**Affiliations:** ^1^Department of Medicine, Clinical Immunology and Hematology, University of Padova, Padova, Italy

**Keywords:** immunoglobulin replacement therapy, secondary hypogammaglobulinemia, chronic lymphocytic leukemia, multiple myeloma, bone marrow transplantation, solid organ transplantation, subcutaneous immunoglobulins

## Abstract

Immunoglobulin (Ig) replacement therapy dramatically changed the clinical course of primary hypogammaglobulinemias, significantly reducing the incidence of infectious events. Over the last two decades its use has been extended to secondary antibody deficiencies, particularly those related to hematological disorders as lymphoproliferative diseases (LPDs) and multiple myeloma. In these malignancies, hypogammaglobulinemia can be an intrinsic aspect of the disease or follow chemo-immunotherapy regimens, including anti-CD20 treatment. Other than in LPDs the broadening use of immunotherapy (e.g., rituximab) and immune-suppressive therapy (steroids, sulfasalazine, and mycophenolate mofetil) has extended the occurrence of iatrogenic hypogammaglobulinemia. In particular, in both autoimmune diseases and solid organ transplantation Ig replacement therapy has been shown to reduce the rate of infectious events. Here, we review the existing literature about Ig replacement therapy in secondary hypogammaglobulinemia, with special regard for subcutaneous administration route, a safe, effective, and well-tolerated treatment approach, currently well established in primary immunodeficiencies and secondary hypogammaglobulinemias.

## Introduction

Immunoglobulins (Ig) have been commercially available since the 1940s, and first carried out as replacement therapy (IgRT) in a patient with agammaglobulinemia in 1951 by Ogden Bruton. From that first experience on, Ig have been widely used in patients with primary immune deficiency (PID), as it is well known that hypogammaglobulinemia is a crucial risk factor for development of infectious events and IgRT is able to reduce this risk. By contrast, the lack of clear indications regarding the use of prophylactic IVIG in secondary antibody deficiencies (SAD) may appear paradoxical, if we consider the broad range of disorders in which hypogammaglobulinemia is an intrinsic aspect of the disease or a iatrogenic consequence (Table 1) and being the number of affected patients significantly higher than in PID.

**Table 1:** **Main causes of secondary hypogammaglobulinemia**.

Secondary hypogammaglobulinemia	
Excessive loss of immunoglobulins	Protein-losing enteropathy
	Nephrotic syndrome
	Severe burns
Malignancy	Chronic lymphocytic leukemia
	Multiple myeloma
	Good’s syndrome
	Non-Hodgkin B cell lymphomas
Drug induced	See Table 2

In this review, we summarize the most common conditions in which SAD can be observed. We also describe evidence indicating the possibility of subcutaneous (SCIG) rather than intravenous (IVIG) Ig for the treatment for SAD.

## Chronic Lymphocytic Leukemia

Infective complications account for up to 50% of all chronic lymphocytic leukemia (CLL)-related deaths (1). Their etiology is multifactorial, due to disease-related immune defects and/or chemo-immunotherapy treatment regimens (2, 3). Hypogammaglobulinemia is the most common chronic immune defect in patients with CLL (the prevalence ranges from 20 to 70%), and correlates with duration and stage of the disease (4). The defect is usually irreversible even in patients achieving complete disease remission. A direct relationship was found between low levels of IgG and the frequency/severity of infections.

Two main strategies are indicated in UK guidelines for patients with hypogammaglobulinemia who develop recurrent bacterial infections (1): antibiotic prophylaxis and IgRT. Nonetheless, there are no standard guidelines for antimicrobial prophylaxis in CLL patients, and most recommendations are derived from small clinical trials and anecdotal reports (4, 5).

Regarding prophylactic IVIG in patients with CLL and hypogammaglobulinemia, randomized controlled studies have been performed in the 1980s ad 1990s and recently summarized (6): they suggest that the use of IVIG may be considered in patients with hypogammaglobulinemia secondary to CLL who experience recurrent infections, since IVIG could significantly decrease the number of infections and the use of antibiotics, reducing hospitalization need and loss of working days. Despite this, the authors did not found any difference in all cause mortality between the treated and control group, maybe due to the relatively short follow-up time (1 year). For these reasons, according to UK guidelines IgRT indicated in patients with a serum IgG < 500 mg/dl complaining of recurrent or severe infections (1). Similar indications are suggested in the Canadian guidelines (7) and by the panel group of the Primary Immunodeficiency Committee of the American Academy of Allergy, Asthma, and Immunology (8). In all these guidelines, an initial dose of 400 mg/kg administered intravenously 3–4 weekly is suggested, aiming at a trough level of 600–800 mg/dl (1, 7). The Ig dose should be then adjusted according to clinical response and steady state trough levels (IgG trough level > 400 mg/dl). As in PID, higher trough levels may be beneficial in patients with underlying co-morbidities, particularly bronchiectasis. On the basis of these evidences and our experience (9), the same indications are followed in our outpatient clinic. We regularly evaluate the response to IgRT after 12 months, to assess its efficacy and to judge the need of continuing or stopping the infusions.

Attempts have been made to define the risk factors for infections in CLL, in order to select patients who could benefit from IgRT even with a pre-emptive approach. In two papers (10, 11), factors other than Ig concentration (previous chemotherapy, clinical stage, CD38 expression, genetic analysis, and IgVH mutations) have been pointed out as the main prognostic markers for the development of infections, while low IgG level seem not to be clearly associated with infections. Nonetheless, in these studies IgG levels were recorded independently from infectious events, at diagnosis or at the moment of the survey. In CLL, hypogammaglobulinemia is commonly progressive over time; thus, IgG levels evaluated at onset of the disease may significantly differ from levels observed at the time of infection onset. Dhalla et al. (12) suggested that immunization responses could be used to stratify infection risk and select patients for IgRT, but caution is recommend since interpreting immunization assays is complex, cut-off levels may vary and there are controversies in stating what constitutes an adequate response. Finally, Freeman et al. (13) suggested that screening patients with CLL for IgG subclass deficiency may be a useful adjunct in stratifying their risk for infection, since they were able to show a significant relationship between any IgG subclass deficiency and infections, regardless of the total Ig level.

## Multiple Myeloma

Infection is a significant cause of morbidity and the leading cause of death in patients with multiple myeloma (MM) (14). The increased susceptibility to infections results from the interplay between antineoplastic therapies and disease-related complications (15). Moreover, novel treatments significantly prolonged survival transforming MM into a chronic condition, characterized by multiple relapses and salvage therapies, resulting in an increased cumulative immunosuppression and a higher risk for infection. The incidence of infectious events varies in different phases of MM (16), and seems to be higher during active disease and in the first months of induction chemotherapy. In early-stage MM, the most common infections involve the respiratory tract, manifesting as bronchitis and pneumonia. These infections are predominantly caused by *H. influenzae* or *S. pneumonia*, suggesting a role of hypogammaglobulinemia in their pathogenesis. In patients with advanced disease and during the neutropenic phases of intensive chemotherapy, *S. aureus* and gram-negative bacteria are more common, thus implying different underlying pathogenic mechanisms.

As in CLL, only few studies evaluated the role of IgRT in patients with hypogammaglobulinemia and MM. Chapel et al. (17) in 1994 performed a randomized, double-blind, placebo-controlled, multicenter trial of IVIG employed as prophylaxis against infection in 82 patients with stable MM; the authors demonstrated a protective role of IVIG against life-threatening infections and a significant role in reducing the risk of recurrent infections. Other two studies have been performed in patients with hypogammaglobulinemia and either CLL or MM, obtaining similar results (6). Conversely, Blombery et al. (18) showed no benefit of the use of peritransplant IVIG to reduce infectious complications in hypogammaglobulinemic patients with MM undergoing autologous stem cell transplantation.

In our opinion, regular Ig substitution should be considered in MM patients who suffer from life-threatening or recurrent infections that are reasonably thought to be caused by low levels of polyclonal Ig (16, 19). Similar indications are suggested in the Canadian guidelines about IgRT (7), where the proposed regimen consists of 400 mg/kg of IVIG administered every 4 weeks, subsequently adjusted to reach an individualized “biological” trough level. Prophylactic therapy with Ig during autologous stem cell transplantation is not recommended. Unfortunately, we observed that it may be not so easy to exactly define the level of polyclonal Ig in patients with MM, due to the presence of the monoclonal protein that interfere with Ig determinations. We suggest that clinical evaluation and anamnestic aspects are fundamental to decide if may be helpful to prescribe or not substitutive therapy in MM.

## Iatrogenic Hypogammaglobulinemia

As shown in Table 2, pharmacological history of the patient should be carefully evaluated when hypogammaglobulinemia is detected.

**Table 2:** **Main causes of drug-induced hypogammaglobulinemia**.

Drug-induced hypogammaglobulinemia	
Anti B cells monoclonal antibodies	Rituximab
	Belimumab
Immunosuppressants and chemotherapeutics	Steroids
	Gold salts
	Azathioprine
TKI inhibitors	Imatinib
	Dasatinib
Anticonvulsants	Carbamazepine
	Valproate
Others (sporadically described)	Ramipril
	Acetylsalicylic acid

One of the most used drug able to induce iatrogenic hypogammaglobulinemia is the anti-CD20 monoclonal antibody rituximab. Firstly experimented and introduced in the clinical practice for the treatment of hematological malignancies, it has become a commonly used immune modulatory strategy for the treatment of many refractory or poorly controlled autoimmune or inflammatory disorders. Removal of CD20-expressing cell populations induces a dysregulation of immune homeostasis, impacting regulatory functions of normal B cells (20, 21). Hypogammaglobulinemia represents common negative consequences of this imbalance (22–27), and is considered the main factor that influences the increased risk of infection in patients receiving rituximab (28–30). According to recent hypotheses (31–33), a subgroup of rituximab-induced hypogammaglobulinemias should be considered as the consequence of the presence of a latent PID. In some cases, rituximab would elicit the antibody defect in genetically predisposed individuals (34).

Kelesidis et al. (35) recently summarized the available evidence about rituximab use and infections: the risk was increased in patients with hematological malignancies, while in patients with autoimmune diseases the risk was similar to other treatments. However, considering the number of confounders potentially masking or modulating the overall effects (e.g., co-morbidities, concurrent chemotherapy, presence of neutropenia, number of rituximab cycles, etc.), caution has been suggested in drawing definitive conclusions. In hematological conditions, the incidence of transient or persistent hypogammaglobulinemia following rituximab-therapy ranges from 15 to 40% (28). Female gender, fludarabine association regimens (36), and administration after autologous stem cell transplantation (37–40) have been identified as main risks factors, while maintenance therapy for follicular lymphoma is associated with a very low incidence of hypogammaglobulinemia (41). Regarding autoimmune disorders, two recent studies described a possible detrimental effects of rituximab on Ig synthesis in patients with ANCA-associated vasculitides (42, 43), and in particular in patients relapsing after cyclophosphamide treatment (43). Conversely, other studies contradict these observations: Marco et al. (44) reported that hypogammaglobulinemia could be attributed to the prior cyclophosphamide/steroid exposure rather than to the cumulative rituximab dose. Finally, the risk of hypogammaglobulinemia and infections seems to be related to the underlying autoimmune condition: in patients with rheumatoid arthritis or immune thrombocytopenia, the treatment is well-tolerated without significant risk of infections and hypogammaglobulinemia (22, 45–47).

We suggest that Ig levels should be always checked before rituximab administration and monitored for at least 6 months after the last dose. A longer period of observation should be addressed in patients presenting with low Ig levels prior to rituximab-therapy or in those who show risk factors for prolonged/severe hypogammaglobulinemia. In any case prompted IgRT should be offered, either e.v. or subcutaneous, to those patients who complain severe or recurrent infection episodes and concomitant low Ig levels.

## Bone Marrow Transplantation

Allogeneic bone marrow transplantation (ASCT) is a well established therapy in hematological malignancies. Immune defects are common consequences, persisting for years after ASCT. Arai et al. (48) recently described that the cumulative incidence of hypogammaglobulinemia (IgG < 400 mg/dl) in a cohort of 278 patients was 24.1 or 27.1%, respectively 1 or 3 years after ASCT. Risk factors were lymphoid malignancies, history of previous ASCT, usage of mycophenolate mofetil, low pre-ASCT IgG levels, and grade 2–4 aGVHD. Norlin et al. (49) described that patients with low IgG levels ( <400 mg/dl) had an increased risk of transplant-related mortality compared to patients with moderately low or normal levels. Patients with low IgG levels had an increased incidence of infections as cause of death.

A recent meta-analysis (6) summarizes data on IgRT in patients undergone ASCT. When polyvalent Ig or hyperimmune cytomegalovirus CMV-IVIG are compared, there are no differences in mortality, rate of infections, CMV infections, and microbiologically documented infections. Polyvalent Ig significantly reduced the risk for interstitial pneumonitis but increased the risk for veno-occlusive disease and adverse events. Moreover, guidelines for preventing infectious complications provided by the American Society of Bone Marrow Transplantation (50) suggest that benefit is small if IVIG are used for prophylaxis of bacterial infections in patients with severe hypogammaglobulinemia (defined by authors as IgG < 400 mg/dL). The panel of experts recommends that IVIG dose and frequency should be individualized to maintain trough serum IgG concentrations >400 mg/dL. Again, IVIG are not recommended for CMV-disease prophylaxis after ASCT. Interestingly, Sundin et al. (51) showed that SCIG therapy is as effective as IVIG in maintaining IgG levels above 400 mg/dl in pediatric patients after ASCT, and SCIG were associated to less adverse events compared to IVIG.

## Solid Organ Transplantation

Hypogammaglobulinemia has been reported as a complication of solid organ transplantation, particularly after heart, lung, and kidney transplantation, with an associated increased risk for infections. Moderate to severe hypogammaglobulinemia in recipients of lung transplantation has been associated with increased occurrence of infections, longer hospitalization, and acute cellular rejection. Hypogammaglobulinemia appeared to be related to 1-year all cause mortality. A recent meta-analysis by Florescu et al. (52) found a consistent prevalence of hypogammaglobulinemia (45%) and severe hypogammaglobulinemia (15%) during the first post-transplant year. The risk of infections was higher (2.46-fold) when IgG were less than 400 mg/dL; the risk of infections in patients with IgG > 400 mg/dL did not differ with respect to patients with normal Ig levels. This risk is related to the immune-suppressive treatment, being particularly high if mycophenolate mofetil is employed.

Concerning the role of IgRT in transplanted patients with hypogammaglobulinemia it has been reported that IgRT favors the reduction of infections rate (53, 54). Moreover, a single center experience (55) suggested that SCIG replacement therapy represents a well-tolerated alternative to IVIG after lung transplantation. Unfortunately, these studies are heterogeneous in terms of study populations or/and criteria of inclusion/exclusion for the beginning of IgRT and definitive conclusion cannot be drawn. Anyway, we suggest to start IgRT in patients with severe hypogammaglobulinemia, or in patients who complain recurrent infections reasonably related to low IgG levels. The proposed starting regimen should be 400 mg/kg of IVIG administered every 4 weeks, subsequently adjusted to reach an individualized “biological” trough level.

## Other Causes of Secondary Hypogammaglobulinemia

Conditions that frequently cause Ig loss include burns, renal, or intestinal diseases. Protein-losing enteropathy is characterized by an excessive loss of proteins into the gastrointestinal tract, resulting in some cases in symptomatic hypogammaglobulinemia. An hypoproteinemic state with hypogammaglobulinemia can be also a consequence of severe burns. Treatment of these disorders consists mainly in the management of the underlying disease, and no extensive data are available regarding IgRT.

A variety of infections has been recognized as an important cause of morbidity and mortality in patients with nephrotic syndrome. Immune defects in these patients are due to edema complications, urinary loss of complement factors and Ig, and defects in cellular immunity and secondary effects of immunosuppressive therapy. Many different prophylactic interventions have been used for reducing the risks of infection in these patients but recommendations for routine use are still lacking (56).

Hypogammaglobulinemia is present in a small percentage of patients with thymoma (Good’s syndrome), usually symptomatic. Often, hypogammaglobulinemia persists after the treatment of the underlying disease. There are some reports on the efficacy of IgRT in these patients (57, 58).

## HIV Infection

HIV infection is peculiar in its immunological profile. Patients usually exhibited hypergammaglobulinemia that encompassed all three Ig classes, but given the T cell defect they failed to mount specific antibody responses to various T cell dependent antigens, and in particular encapsulated bacteria (59). In adults with HIV, opportunistic infections are the most common manifestations of the immune deficiency; instead, children frequently experience a different immunological impairment, complaining of recurrent serious bacterial infections (bacteremia and pneumonia) (60).

On the basis of the results obtained in the NICHHD trials in 1991 (61) and several smaller studies previously performed, IVIG have been approved for preventing invasive bacterial infections in children with HIV infection (400 mg/kg every 2–4 weeks). IVIG seems to have only limited therapeutic benefit in terms of mortality reduction in the acute phase of the bacterial infections (60). The utility of IgRT has been later questioned, because antibiotic prophylaxis and especially retroviral therapy have dramatically changed the clinical course of the disease (60); nowadays IgRT is taken into account only in selective cases with recurrent bacterial infections despite antiretroviral and antibiotic therapy.

Adult patients with HIV may retain their full complement of B cells, and are less susceptible to infections with common pathogens, even if HIV can profoundly affect humoral immunity in a subset of patients. Several uncontrolled trials did not shown conclusive benefits to routine IgRT (59). These studies should not exclude the use of IVIG for adults who are infected with HIV-1 with recurrent bacterial infections despite antiretroviral and antibiotic therapy.

## Subcutaneous Immunoglobulins

Subcutaneous Ig have been widely shown to be safe, cost-effective, and greatly appreciated in terms of Health-Related Quality of Life (HRQL) in patients with PID (62–66). SCIG can be self administered at home, do not require venous access and systemic premedication, are characterized by a gradual absorption of the drug and by a decrease in the incidence of systemic adverse effects (66, 67). Local reactions are usually mild and do not affect the good tolerability of the treatment (68).

Safety and efficacy of SCIG in SAD have been assessed in few case series. We showed that SCIG are safe and effective in maintaining adequate levels of serum IgG in patients with lymphoproliferative diseases (LPDs) and hypogammaglobulinemia (9). We also observed an improvement perceived in HRQL. Similar results in term of efficacy, safety, and HRQL were obtained in children after hematopoietic stem cell transplantation (51) and in patients after lung transplantation (55).

Other potential benefits of SCIG should be considered in patients with hematological malignancies. Venous access often represents a great concern after chemotherapy treatments, and SCIG provide the possibility to avoid the use of venous accesses. The flexibility of SCIG treatment and the possibility of a home-based infusion represent a further advance for patients who usually need an high number of outpatient visits.

## Open Questions

In this review, evidence has been provided suggesting that IgRT is effective in most patients with SAD. Nonetheless, cost-effectiveness advantage of initiating an IgRT in subjects with SAD has been criticized by some authors. To provide a conclusive reply to this criticism, a question should be definitively answered by future studies: what criteria should be used to identify patients in whom prophylactic IgRT may help to decrease infectious risk? At present IgG levels represent the only parameter employed to this aim. A better selection of patients in which IgRT is needed can lead us to limit the perhaps too extended use of IgRT in SAD.

## Author Contributions

All the authors participate in the definition of the work. Nicolò Compagno, Giacomo Malipiero, and Francesco Cinetto prepared the manuscript. Carlo Agostini coordinated the work and critically revised the manuscript. The final version of this work was approved by all authors.

## Conflict of Interest Statement

Carlo Agostini received research grants from CSL Behring and Baxter. The other authors declare that they have no relevant conflicts of interest.
